# Walking with a Passive Hip Exoskeleton and Wearables: Gait Characteristics and Metabolic Power in Senior Adults

**DOI:** 10.3390/s26010100

**Published:** 2025-12-23

**Authors:** Cristina-Ioana Pîrșcoveanu, Pascal Madeleine, Ernst Albin Hansen, Jesper Franch

**Affiliations:** 1 Department of Health Science and Technology, ExerciseTech, Aalborg University, 9220 Aalborg, Denmark; civ@hst.aau.dk (C.-I.P.); jfranch@hst.aau.dk (J.F.); 2 Centre for Health and Rehabilitation, University College Absalon, 4200 Slagelse, Denmark; ernsthansen69@gmail.com

**Keywords:** IMU, cadence, step length, exoskeleton, metabolic power, seniors

## Abstract

**Background**: This study explored the potential of a passive exoskeleton (Exo) to improve cadence, step length, oxygen uptake, and reduce metabolic power in senior adults, with the expectation that slow walkers (SW < 0.56 m/s) would benefit more than intermediate walkers (IW ≥ 0.56 m/s). **Methods**: Twenty-three senior adults walked on a treadmill at their self-selected speed using the Exo, noExo, and a placebo (Sham) in a randomized and balanced order. A lower back inertial measurement unit, a heart rate monitor, and an oxygen uptake system were used to monitor spatiotemporal and cardiopulmonary parameters. Cadence, step length, heart rate, oxygen uptake (VO_2_ and relative VO_2_), metabolic power, and respiratory exchange ratio were extracted. A two-way MANOVA was performed across Exo vs. noExo vs. Sham and SW vs. IW. **Results**: Using Exo did not show any significant changes in spatiotemporal or cardiopulmonary outcomes compared to the conditions for both SW and IW. IW vs. SW seniors had significantly higher cadence (15–19%), step length (31–41%), relative VO_2_ (21–23%), and metabolic power (21–23%) in all devices (*p* < 0.05). **Conclusions**: These findings show that the use of Exo among senior adults does not improve spatiotemporal parameters nor reduce metabolic powers even among SW.

## 1. Introduction

Age-related decline in physical activity is a global issue that often leads to sedentary behavior. Physical inactivity has a large impact on shortening average life expectancy [1] and contributes to further metabolic disorders in diabetic adults [2,3]. Consequently, “successful aging” [4,5] has gained popularity by integrating regular physical and mental activities into the daily lives of the senior adult population [6,7]. Walking assistive devices, particularly wearable exoskeletons, are increasingly being investigated as tools for augmenting functional capacity in senior adults [7,8,9,10]. Exoskeletons represent an emerging class of walking aids designed to enhance mobility by providing support to single or multiple joints and augmenting the natural joint torques [11,12,13,14,15]. These devices may employ active actuators [7] or passive elastic spring-based elements [16] and are used to compensate for age-related deterioration of the neuromusculoskeletal system, often characterized by increased step variability and metabolic power, reduced walking speed, and step length [11,17]. Additionally, reduced metabolic power has been reported during symmetric repetitive lifting when using passive exoskeletons [18].

Passive single-joint exoskeletons are valued for their simplicity and low weight (<1000 g) and are predominantly hip joint assistive [13,14,15,19,20,21,22]. Exoskeletons have been tested across diverse populations, including asymptomatic adults, senior adults, and seniors with neurological or orthopedic impairments. Most studies investigating passive hip exoskeletons report average walking speeds between 1.1 m/s and 1.5 m/s [13,14,15,19,20,21,22] and sample sizes from single prototype users [20,23] and case studies [24] to larger groups of up to 48 participants [22]. Key findings from studies of such devices include increased walking distance, speed [14], and enhanced gait parameters such as hip flexion, toe clearance, and step length [14,15], as well as a reduction in metabolic power ranging from 3.3% [13] to 7.2–8.5% [19,25] in young adults. To evaluate metabolic power when new technologies are explored, a valid and precise cardiopulmonary exercise test system with relevant O_2_, CO_2_, and flow sensors is needed. Furthermore, to connect human movement to metabolic power, the use of accelerometers has previously been used in our lab when evaluating optic flow and thermal imaging in a wide range of walking and running speeds as outcomes for metabolic power [26]. Self-selected walking speed provides vital information on how senior adults move in daily life and allows for the presence of natural variability in walking parameters across different contexts [27,28]. The effect of passive hip exoskeletons on metabolic power has only been investigated at fixed treadmill speeds, specifically 1.1 and 1.5 m/s, without giving participants the possibility for self-selecting a preferred speed [13,19]. This range, although considered within normal ranges, could be classified as “very fast” for most senior women and men, exceeding the cut-off points used to define ‘fast’ (>0.75 m/s) walking speed in gait research [29,30]. The narrow range of fixed walking speeds examined leaves an important gap in understanding how aging, without additional pathologies, is mitigated using exoskeletons across slower walking speeds and whether slow walkers would benefit to a higher degree from these types of devices. Therefore, the potential benefits of exoskeleton assistive devices for walkers preferring slow and intermediate walking speeds remain inadequately explored. This study aimed to evaluate the effects of a passive-assistive, lightweight exoskeleton using both a triaxial inertial measurement unit (IMU) and a cardiopulmonary exercise test system in senior adults.

In agreement with former studies, the use of the passive hip exoskeleton is hypothesized to reduce cadence, increase step length [15], and decrease metabolic power in senior adults [13], with effects being influenced by the self-selected walking speed. Feodoroff and Blümer [15] did not measure metabolic power but identified spatiotemporal changes at lower walking speeds compared to Panizzolo et al. [13] who found improvements in metabolic power without changes in spatiotemporal parameters. We therefore expected that participants walking with the exoskeleton at lower walking speeds would lower metabolic power and change spatiotemporal parameters, whereas participants walking at higher speeds would maintain spatiotemporal parameters at lower metabolic power. In general, walking at higher speeds increases oxygen uptake [26]; therefore, higher metabolic cost is expected in participants walking at higher speeds.

## 2. Materials and Methods

Twenty-three senior adults (21 females and 2 males aged 72.7 ± 4.6 years; height 1.67 ± 0.07 m; body mass 73.0 ± 13.7 kg) participated in this study. All participants were >65 years, capable of walking on a treadmill for more than 10 min and presented no uncorrected visual or vestibular deficits. No caffeine intake or physical activity was allowed for a minimum of 3 h before the test session [31]. The participants’ level of frailty, physical activity, and quality of life were evaluated using the Tilburg Frailty Index, the International Physical Activity Questionnaire (IPAQ), and EQ-5D-5L, respectively [32,33]. Written informed consent was given by all participants, and the study was conducted in accordance with the ethics committee of the North Denmark Region (LBK no: 1083) and the Helsinki Declaration.

The participants were asked to walk at their preferred speed on a motor-driven treadmill (Woodway Pro XL; Woodway Inc., Waukesha, WI, USA). This speed was determined prior to testing while walking without an exoskeleton and then kept constant across all three randomized, balanced conditions: no exoskeleton (NoExo), bilateral passive hip exoskeleton (Exo), and bilateral sham device (Sham). The passive hip exoskeleton (aLQ, IMASEN Electrical Industrial Co., Ltd., Aichi, Japan) used in this study is a bilateral spring-based system and has a total weight of approximately ~1000 g located on the lateral side of each hip (~480 g each) [21,22]. The device is available in three sizes to accommodate different body heights and weights. During walking, the exoskeleton functions like a pendulum—loading the springs during hip extension and releasing energy during hip flexion [15] and delivering an assistive movement ranging from 0 to 2.8 Nm over a hip joint range of motion of 0 to 84° [21,22]. The Sham device retained the shape and weight of the original device but had the spring mechanism disabled.

**Figure 1:**
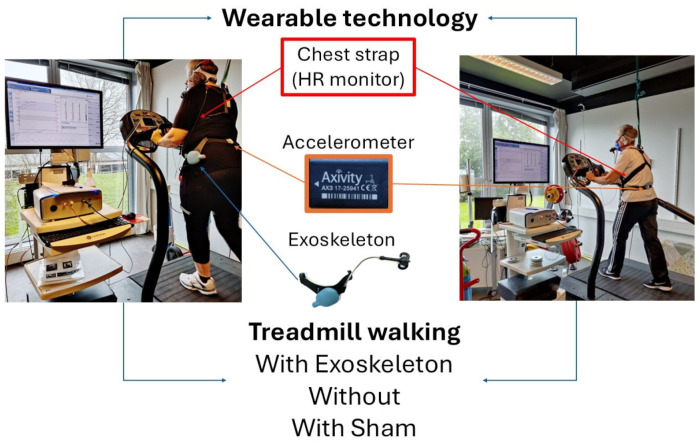
Illustration of experimental design of two senior adults walking on a treadmill with and without exoskeleton and with a sham; their oxygen uptake was monitored by Vyntus and their spatiotemporal parameters and heart rate were monitored using wearable technology, i.e., an IMU and a heart rate monitor, respectively.

All participants had prior exposure to both the exoskeleton and the sham device, as they had previously taken part in two related studies, thereby accumulating more than two hours of user experience [21,22]. The average overground walking speed from the previous studies was used as a starting point to determine the self-selected walking speed of each participant (higher or lower). Additionally, before testing, a 10 min familiarization walk on the treadmill was allocated to all participants. Each walking condition was 7 min long, separated by a 3 min rest. An IMU (AX3, Axivity, Newcastle, UK) was fitted to the lower back at the fifth lumbar vertebrae, sampling at 100 Hz [34]. Before the start of each device condition, the IMU was tapped three times to determine the change in the vertical acceleration and detect the start of each condition (i.e., Exo, noExo, and Sham). Heart rate was measured continuously with a chest strap (Polar RCX5 GPS system, Kempele, Finland). An automated online breath-by-breath system (Vyntus CPX, Vyaire Medical, Mettawa, IL, USA) was used to measure pulmonary VO_2_ and VCO_2_ during the 7 min treadmill walk in each condition (Figure 1). Before data collection, the volume transducer (Triple V) and gas analyzers were calibrated with an internal flow generator (12–120 L/min), a calibration gas of known concentration (O_2_% = 15.00%; CO_2_% = 5.00%), and ambient air (O_2_% = 20.93%; CO_2_% = 0.03%), respectively. Ventilatory data are reported at standard (STPD) conditions.

The IMU data was downloaded through the OpenMovement GUI application and imported into MATLAB^®^ (MathWorks, Release R2023b v23, Natick, MA, USA). A pre-validated gait detection algorithm from the Mobilise-D consortium [35] adapted to our dataset, which detrends filters using a fourth-order Butterworth filter with a cutoff frequency of 20 Hz, and smooths the data that is used [21]. Cadence, step length, and walking speed were extracted from data corresponding to the last two minutes (5:00–7:00). The participant’s order of the conditions and height were imported separately using an Excel file (Microsoft 365 Apps v2110, Redmond, WA, USA) to segment and reorder the data into the three assessed conditions, as well as to adjust the algorithm to each participant. The cadence, step length, and walking speed were computed as twice the stride frequency, half of the distance between two non-consecutive initial contacts [35], and the product of cadence and step length divided by 60, respectively [35,36]. Averaged heart rate and breath-by-breath data at minute 5:00–7:00 were used as a response to each condition. VO_2_, VCO_2_, and respiratory exchange ratio (RER) were used to quantify the metabolic power of each exoskeleton condition [37]. Furthermore, the relative VO_2_ and metabolic power were normalized to the individual’s body mass. To separate metabolic effects of device mechanics from those that were caused solely by added load, these two parameters were also normalized to the carried weight (830–840 g without the waist belt—which was worn in all three device conditions). This allowed us to assess whether the weight of the exoskeleton increased metabolic power beyond the natural energetic cost of walking.

Walking speed was used to divide the senior adults into two subgroups: slow walkers (SW) < 0.56 m/s (11 females and 2 males with an average walking speed 0.47 ± 0.12 m/s) and intermediate walkers (IW) ≥ 0.56 m/s (10 female participants with an average walking speed 0.66 ± 0.31 m/s). Only two participants had a speed above 1 m/s and were included in the intermediate group. The cut-off point in walking speed was selected using the median value and is comparable to the literature cut-off point of 0.4–0.6 m/s [29].

All statistical tests were performed using IBM SPSS Statistics (v29, IBM SPSS^®^ Statistics, Armonk, NY, USA). Descriptive statistics were reported as mean and standard deviation. The normality of data was assessed using Q-Q plots and box plots and the Shapiro–Wilk test [38]. Two-way multiple analysis of variance (MANOVA) was conducted for cadence, step length, HR, VO2, relative VO2, RER, with metabolic power for all devices (Exo vs. noExo vs. Sham) and walking speed (SW vs. IW) as fixed factors. In case of significance, corrected Bonferroni post hoc analysis was performed for pair-wise comparisons. Partial eta squared (η_p_^2^) for each parameter was extracted, where a large effect size [39] was considered if η_p_^2^ ≥ 0.026, a moderate effect size was considered if η_p_^2^ ≥ 0.13, and a small effect size if η_p_^2^ ≥ 0.02. A significant alpha level was set as *p* < 0.05.

## 3. Results

All parameters were normally distributed, except for step length during noExo (*p* = 0.048). However, when examining the Q-Q plots, the data indicated normality, so no further action was taken. Table 1 provides an overview of the two subgroups’ descriptive demographic and anthropometric data.

The MANOVA showed an overall significant difference on all the combined dependent parameters, except for absolute VO2 between SW and IW groups (Wilks Λ = 0.44, F(7,57) = 10.36, *p* < 0.001, η_p_^2^ = 0.56). No significant differences were seen for the device used or interaction of device × speed on the combined dependent parameters (Table 2).

**Figure 2:**
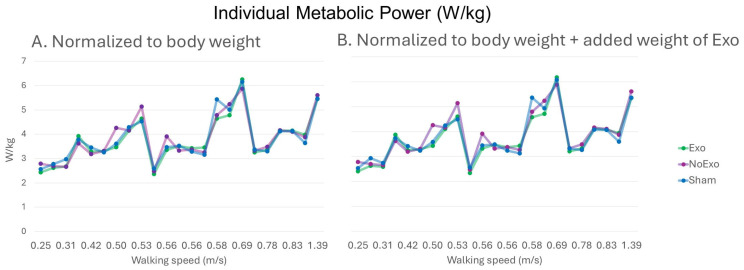
Comparison of individual metabolic power in relation walking speed (**A**)—without and (**B**)—with normalization to exoskeleton weight.

**Figure 3:**
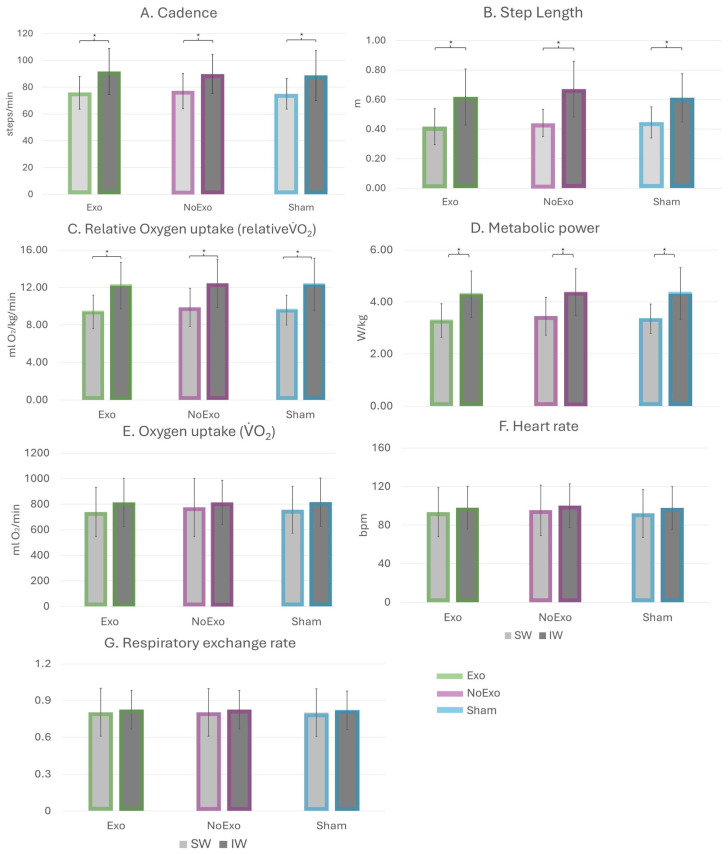
Bar plots of dependent parameters: (**A**) Cadence, (**B**) Step length, (**C**) Relative Oxygen Uptake (normalized for bodyweight + exoskeleton weight), (**D**) Metabolic power (normalized for bodyweight + exoskeleton weight), (**E**) Oxygen uptake, (**F**) Heart rate, and (**G**) Respiratory exchange rate expressed as mean ± standard deviation. Light gray bars illustrate slow speed walkers (SW), and dark gray bars illustrate intermediate speed walkers (IW) for Exo (green), NoExo (purple), and Sham (blue). Significant differences are illustrated with the * (corrected Bonferroni post hoc analysis).

Figure 2 shows the normalized individual metabolic power without and with normalization to exoskeleton weight in relation to walking speed. The corrected post hoc analysis specified that the walking speed significantly influenced cadence, step length, relative VO_2_, and metabolic power in all device conditions (Figure 3A–D). Cadence was significantly higher in IW vs. SW across all device conditions (Exo: 91.73 ± 17.27 vs. 75.79 ± 12.11, *p* = 0.02; NoExo: 89.79 ± 14.68 vs. 77.03 ± 13.09, *p* = 0.05; Sham: 88.78 ± 18.56 vs. 75.03 ± 11.35, *p* = 0.03; Figure 3A). Similarly, step length was significantly higher in IW vs. SW across all device conditions (Exo: 0.62 ± 0.19 vs. 0.42 ± 0.12 m; NoExo: 0.67 ± 0.19 vs. 0.44 ± 0.09 m; Sham: 0.61 ± 0.16 vs. 0.45 ± 0.11 m; *p* = 0.01; Figure 3B). Relative VO_2_ normalized for bodyweight + exoskeleton weight was significantly higher in IW vs. SW across all device conditions (Exo: 12.19 ± 2.49 vs. 9.39 ± 1.80 mLO_2_/kg/min; NoExo: 12.43 ± 2.59 vs. 9.87 ± 2.06 mLO_2_/kg/min; Sham: 12.30 ± 2.80 vs. 9.59 ± 1.59 mLO_2_/kg/min; *p* = 0.01; Figure 3C). In addition, metabolic power normalized for body weight + exoskeleton weight was significantly higher in IW vs. SW across all device conditions (Exo: 4.29 ± 0.90 vs. 3.28 ± 0.64 W/kg; NoExo: 4.37 ± 0.91 vs. 3.45 ± 0.73 W/kg; Sham: 4.32 ± 0.99 vs. 3.35 ± 0.57 W/kg; *p* = 0.01; Figure 3D). Almost similar results were observed in relative VO_2_ (IW vs. SW-Exo: 12.35 ± 2.54 vs. 9.49 ± 1.81 mLO_2_/kg/min; Sham: 12.46 ± 2.85 vs. 9.70 ± 1.60 mLO_2_/kg/min) and metabolic power (IW vs. SW-Exo: 4.35 ± 0.91 vs. 3.32 ± 0.65 W/kg; Sham: 4.38 ± 1.01 vs. 3.39 ± 0.57 W/kg) when both parameters were normalized only to bodyweight. No significant differences were observed for HR, VO_2_, and RER (Figure 3E–G).

## 4. Discussion

This study aimed to evaluate the effects of wearing a passive-assistive, lightweight exoskeleton on senior adults’ spatiotemporal and metabolic parameters. The findings revealed that, independent of the device used (Exo vs. NoExo vs. Sham), spatiotemporal and metabolic parameters were not significantly altered during treadmill walking at preferred walking speed. Therefore, the results did not support the proposed hypothesis of improved spatiotemporal parameters or reduced metabolic power with exoskeleton use. Further, these outcomes were not affected by self-selected walking speed. Notably, the added weight of the device did not produce additional metabolic power beyond the natural metabolic power of walking, observed through the lack of interaction between NoExo and Sham conditions.

The lack of significant differences between the devices (Exo, NoExo, and Sham) indicated that wearing the current passive exoskeleton does not significantly alter the natural treadmill walking pattern of senior adults. Although passive exoskeletons have been shown to alleviate the physical demands of walking and enhance walking quality [13,15,22] and/or walking endurance [14], the exoskeleton used in this study did not produce significant changes in spatiotemporal or metabolic parameters in asymptomatic active senior adults. Individual metabolic power did not show any discernible trend regarding possible walkers that could benefit to a higher degree. This discrepancy may be attributed to differences in device design (including torque level), targeted joints, participant characteristics, and walking conditions. Feodoroff and Blümer [15] found significant improvements in step length, hip flexion, and toe clearance in neurologic and orthopedic seniors using the same type of device, but only unilaterally. The same exoskeleton was used bilaterally by Pîrșcoveanu et al. [22] in both senior and younger adults, showing no significant changes in basic walking parameters but demonstrating a mitigating effect during more demanding tasks, such as dual-task walking. Panizzolo et al. [13] showed improved metabolic power during treadmill walking of senior adults using an elastic-based bilateral passive hip exoskeleton. The metabolic power was investigated at three different levels of torque (0.3 Nm/kg, 0.5 Nm/kg, and 0.7 Nm/kg), and a significant average reduction of 3% was found in eight out of nine senior adults compared to free walking [13]. Interestingly, each senior achieved his/her lowest metabolic power at different torque levels, with most of them showing a preference for the 0.3–0.5 Nm/kg torque level. Furthermore, in the conditions where participants exhibited the highest metabolic reduction, no statistically significant changes were found in spatiotemporal parameters compared to free walking [13]. The exoskeleton utilized by the present study supports the hip joint shares similarities with the exoskeleton employed by Panizzolo et al. [13] However, it delivers a lower torque profile of 0–0.03 Nm/kg compared to the 0.3–0.7 Nm/kg in the Panizzolo et al. study, limiting the likelihood of observing statistical differences. This underlines that higher levels of torque may be necessary to achieve measurable and meaningful improvements in spatiotemporal parameters and metabolic power in senior adults. Moreover, longer wearing time may be needed to induce tangible changes in gait spatiotemporal parameters and metabolic power [40].

Using a walking speed above >0.56 m/s, as preferred by IW, gives a 15–19% higher cadence and a 31–41% longer step length. Both the relative VO_2_ and the metabolic power were 21–23% higher in IW compared to SW. These differences were unaffected by the exoskeleton used (Exo, NoExo, or Sham). The substantial difference in step length and cadence was expected, as their combined effect determines walking speed [8]. There were no changes in metabolic power, which is in line with Diamond-Ouellette et al. [41] but contrary to Panizzolo et al. [13]. Regardless of which exoskeleton was used, both metabolic power and relative VO_2_ were higher in the IW group. The higher walking speed was also reflected as a main effect in higher RER values of 0.03 by the IW group (Table 1), indicating that the IW group favors glucose oxidation to a slightly greater extent than free fatty acid oxidation. However, this difference in RER values did not affect the interrelationship between VO_2_ and metabolic power between groups and devices. The relative VO_2_ values found in the current study for IW are comparable to unimpaired +40 years asymptomatic adults walking at 1.3 m/s [7]. Furthermore, the metabolic power observed (2.36 to 6.26 W/kg) aligns with values previously reported, such as 3.25 W/kg during overground walking at 1.27 m/s [42], 3.47 W/kg during treadmill walking at 1.25 m/s [8], and 3.5 W/kg during treadmill walking at 0.86 m/s [2].

The exclusive use of treadmill-based measurements may influence the measured outcomes, as previous studies have shown that both young and senior adults tend to self-select significantly lower walking speeds on a treadmill compared to overground walking, influencing spatiotemporal parameters [43]. Although all measurements were conducted using a consistent testing environment (same treadmill and staff), which allows for valid comparisons of metabolic power differences between conditions, Anders et al. [44] showed elevated level of electromyographic activation of gluteal muscle during treadmill vs. overground walking, particularly at a slow walking speed. Therefore, a controlled environment, such as treadmill walking, may attenuate or mask a possible exoskeleton-related effect that could otherwise emerge during non-restricted overground slow walking. Pirscoveanu et al. [22] saw a mitigating effect of the exoskeleton on cadence during dual-tasking situations. However, a unilateral version of the same device was assessed by Federoff and Blümer [15] during treadmill walking for orthopedic and neurologic patients, where the participants showed significantly increased step lengths and reduced cadences. Ultimately, the exoskeleton effects may depend more on requested level of assistance and the targeted population than the environment, highlighting the need to examine neurologic, orthopedic, and other mobility-impaired groups in future work.

A limitation of the current study is the relatively small sample size which reduced the statistical power of our analyses. To document this, we performed post hoc power calculations for each dependent variable using the observed η_p_^2^. Consistent with the very small effect sizes for the device and interaction effects (η_p_^2^ < 0.01), the corresponding post hoc power (1 − β) was low (<0.18), indicating a high likelihood of Type II error. On the contrary, the post hoc power for the speed effect was >0.91, indicating adequate sensitivity for medium-to-large effects. However, smaller effects may have gone undetected, so the non-significant device results should be interpreted cautiously. Larger future samples will be needed to clarify whether subtle device-related changes exist. Another limitation of the current study is the sex imbalance, with 21 females and 2 males, which restricts the generalization of our findings to the broader population. Although women have a lower resting metabolic rate compared to men [45] it is unlikely that this difference would affect the outcomes, as more severe training interventions, causing changes in oxygen uptake, have previously been shown to be independent of sex [46]. Still, sex-related differences in spatiotemporal parameters have been reported in senior adults [47]. Therefore, the findings of the present study should be interpreted as reflective of female walking characteristics and metabolic power.

Mounting wearables on the user [48,49] or device contributes to the objective assessments of motor performance or extent of use of, e.g., occupational devices [50,51,52]. In our study, the combined use of both IMU and cardiopulmonary exercise test equipment provides a broader perspective on gait and metabolic adaptations across asymptomatic seniors. Furthermore, using portable oxygen uptake equipment in the field, as performed during field running [53,54], combined with wearable technology, such as IMU, into passive exoskeletons may facilitate active monitoring and provide feedback to the users regarding both walking characteristics and device usefulness.

## 5. Conclusions

Contrary to our hypothesis, the present investigation demonstrated that wearing a passive hip exoskeleton did not significantly change cadence, step length, relative VO_2_, and metabolic power during treadmill walking, independent of the preferred walking speed used by the senior adults.

## Figures and Tables

**Table 1:** Slow (SW) and intermediate walkers’ (IW) descriptive characteristics.

	SW	IW
**Age (years)**	74.1 ± 4.9	70.8 ± 3.4
**Height (m)**	1.67 ± 0.07	1.67 ± 0.06
**Body mass (kg)**	77.5 ± 10.9	67.0 ± 14.6
**IPAQ (MET-min/week)**	2274 ± 1175	4094 ± 3093
**Frailty**	4 ± 3	3 ± 2
**EQ5D-5L self-rated health status**	76 ± 18	73 ± 20

Abbreviations: IPAQ—international physical activity questionnaire, MET—metabolic equivalent of task, EQ5D-5L—health questionnaire.

**Table 2:** Results of between-subjects for walking speed effect, device effect, and interaction effect on the dependent parameters (cadence, step length, HR, VO_2_, relative VO_2_, RER, and metabolic power), where bold-texted values underline significant differences.

	Speed	Device	Device × Speed
**Cadence** (steps/min)	**F(1,63) = 14.88,** ***p* < 0.01, η_p_^2^ = 0.19**	F(2,63) = 0.10, *p* = 0.91, η_p_^2^ < 0.01	F(2,63) = 0.07, *p* = 0.94, η_p_^2^ < 0.01
**Step Length** (m)	**F(1,63) = 29.57,** ***p* <0.01, η_p_^2^ = 0.32**	F(2,63) = 0.40, *p* = 0.67, η_p_^2^ < 0.05	F(2,63) = 0.27, *p* = 0.76, η_p_^2^ < 0.01
**HR** (beats/min)	**F(1,63) = 8.36,** ***p* < 0.01, η_p_^2^ = 0.12**	F(2,63) = 0.16, *p* = 0.85, η_p_^2^ < 0.01	F(2,63) = 0.01, *p* = 0.99, η_p_^2^ < 0.01
**VO_2_** (mLO_2_/min)	F(1,63) = 1.40, *p* = 0.24, η_p_^2^ = 0.02	F(2,63) = 0.04, *p* = 0.96, η_p_^2^ < 0.01	F(2,63) = 0.04, *p* = 0.96, η_p_^2^ < 0.01
**Relative VO_2_** (mLO_2_/kg/min)	**F(1,63) = 22.68,** ***p* < 0.01, η_p_^2^ = 0.27**	F(2,63) = 0.06, *p* = 0.95, η_p_^2^ < 0.01	F(2,63) = 0.11, *p* = 0.90, η_p_^2^ < 0.01
**RER**	**F(1,63) = 6.73,** ***p* = 0.01, η_p_^2^ = 0.10**	F(2,63) = 0.20, *p* = 0.82, η_p_^2^ < 0.01	F(2,63) = 0.15, *p* = 0.86, η_p_^2^ < 0.01
**Metabolic power** (W/kg)	**F(1,63) = 23.13** ***p* <0.001, η_p_^2^ = 0.27**	F(2,63) = 0.05, *p* = 0.95, η_p_^2^ <0.01	F(2,63) = 0.02, *p* = 0.98, η_p_^2^ <0.01

Abbreviations: HR—heart rate, RER—respiratory exchange rate, min—minute, m—meter, mL—milliliter, kg—kilogram, W—watt, *p*—*p*-value, η_p_^2^—partial eta squared.

## Data Availability

Data available upon request.

## Abbreviations

The following abbreviations are used in this manuscript:

Exo	Exoskeleton
NoExo	Without the exoskeleton
Sham	A placebo version of the exoskeleton
IMU	Inertial measurement unit
IW	Intermediate walkers
SW	Slow walkers
VO_2_	Volume of oxygen
VCO_2_	Volume of dioxide carbon
IPAQ	International Physical Activity Questionnaire
MET	Metabolic equivalent of task
EQ5D-5L	Health questionnaire
HR	Heart rate
RER	Respiratory exchange rate
η_p_^2^	Partial eta squared
*p*	*p*-value
MANOVA	Multiple analysis of variance
